# Lecanemab in practice: AI-derived MRI predictors of benefit and Amyloid Related Imaging Abnormalities (ARIA)

**DOI:** 10.1186/s13195-026-02127-z

**Published:** 2026-06-29

**Authors:** Noa Bregman, Nuno Pedrosa de Barros, Talya Nathan, Mori Hay Levy, Diana Sima, Simon Van Eyndhoven, Aya Bar-David, Orna Aizenstein, Dana Niry, Lilian Atlan, Anan Abu Awad, Elissa Ash, Nurit Omer, Tamara Shiner

**Affiliations:** 1 Cognitive Neurology Unit, Neurological Institute, Tel Aviv Sourasky Medical Center, 6 Weizmann St, Tel Aviv, 6423906 Israel; 2 Department of Neurology and Neurosurgery, Gray Faculty of Medical & Health Sciences, Tel Aviv University, Tel Aviv, Israel; 3 Sagol School of Neuroscience, Tel Aviv University, Tel Aviv, Israel; 4 icometrix, Leuven, Belgium; 5 Department of Radiology, Tel Aviv Sourasky Medical Center, Tel Aviv, Israel

## Abstract

**Introduction:**

Lecanemab, a monoclonal antibody targeting amyloid beta, has demonstrated meaningful clinical benefits in early Alzheimer’s disease (AD), yet real-world data is needed to optimize patient selection and enhance safety monitoring, particularly with respect to amyloid-related imaging abnormalities (ARIA). Integration of quantitative and AI-derived MRI biomarkers may improve risk stratification and prediction of clinical trajectory.

**Methods:**

We conducted a retrospective real-world study of eighty-two patients with biomarker-confirmed early AD who initiated lecanemab at Tel Aviv Sourasky Medical Center between November 2023 and June 2025. Baseline MRI included volumetric T1-weighted imaging and susceptibility-weighted imaging (SWI). Automated whole-brain, regional cortical, and hippocampal volumes, and percentiles were extracted using FDA-cleared AI tools (icobrain by icometrix). Microhaemorrhage (MH) burden was assessed by both human and AI-assisted reads. Cognitive outcomes were evaluated using change in Mini-Mental State Examination (MMSE). Linear regression models assessed MRI predictors of cognitive response, and multivariable logistic regression identified predictors of ARIA.

**Results:**

Patients exhibited significantly lower cerebral volumes at treatment initiation. Mean whole brain percentile, mean gray-matter (GM) percentile, and mean white matter percentile were 11.45%, 8.6% and 38% respectively. Higher baseline GM volume predicted less MMSE decline at 12 months (β = 0.64, FDR-corrected p < 0.003). Hippocampal and white-matter volumes were not associated with cognitive outcomes. Seventeen patients (20.7%) developed ARIA. Baseline MH burden was the strongest predictor of ARIA (human rated OR=3.48 per MH, p=0.015, icobrain rated OR=3.25, p=0.01), while APOE ε4 carriage showed a strong directional trend which did not reach significance. Aspirin use and hypertension were not associated with ARIA. Agreement between icobrain and experts for MH ratings was excellent with a single-measure intraclass correlation coefficient (ICC) of 0.89 (95% CI: 0.83–0.93).

**Conclusions:**

AI-derived MRI markers, particularly GM volume and MH burden, provide valuable predictors of cognitive response and ARIA risk in patients treated with lecanemab. Integrating quantitative neuroimaging into clinical workflows may enhance personalized treatment decisions and improve real-world implementation of Amyloid-targeting therapies.

## Introduction

Amyloid-targeting therapies (ATTs) represent a breakthrough in the treatment of early Alzheimer’s disease (AD). Lecanemab, a monoclonal antibody that selectively binds soluble Aβ protofibrils, demonstrated significant clinical and biomarker benefits in the CLARITY-AD phase 3 trial [1], leading to regulatory approval in the United States, Israel, Japan, Europe, and multiple additional regions. As lecanemab becomes increasingly adopted in clinical practice, real-world data is essential to understand treatment efficacy, optimize patient selection, and monitor safety outcomes in the heterogeneous populations seen outside controlled clinical trials [2–5]. One of the major challenges in implementing ATTs is the risk of amyloid-related imaging abnormalities (ARIA), including ARIA-E (vasogenic edema) and ARIA-H (microhaemorrhages and superficial siderosis) [1, 4, 6–8]. Although most ARIA events are asymptomatic or mild, they require careful monitoring and at times require treatment interruption [2, 6–8].

Baseline MRI is therefore a critical component of eligibility assessment, providing information on cerebrovascular pathology, brain atrophy patterns, and other structural features that may influence both safety and clinical outcomes during treatment.

Despite the significant role of MRI in ATT workflows, evidence describing how specific baseline imaging characteristics relate to clinical outcomes and ARIA risk in real-world settings remains limited. Clinical trials have identified associations between APOE ε4 carrier status, microhaemorrhage (MH) burden, and ARIA incidence [1], but less is known about how regional cortical volumes, hippocampal volume, ventricular enlargement, or other quantitative MRI markers influence treatment trajectories in routine practice [9, 10]. Moreover, the generalizability of trial findings to diverse patient populations, different MRI protocols, and real-world clinical decision pathways remains uncertain.

We incorporated advanced AI-based neuroimaging tools via a collaboration with icometrix (icometrix NV, Leuven, Belgium). The company provides FDA-cleared software for quantitative brain MRI analysis, including automated detection and quantification of white-matter lesions, MH, and other biomarkers relevant to neurodegeneration. Automated extraction may support standardized, reproducible, and scalable assessment of structural changes while reducing inter-reader variability and improving sensitivity to subtle pathological patterns.

The Cognitive Neurology Unit at Tel Aviv Medical Center (TASMC) is among the earliest centres outside the United States to integrate lecanemab into routine practice and currently manages a growing real-world cohort within structured clinical workflows supported by quantitative neuroimaging and systematic safety monitoring [2]. Here, we report baseline MRI characteristics in patients treated with lecanemab at the TASMC and examine associations with clinical outcomes and ARIA risk, providing real-world insights into the utility of quantitative and AI-derived MRI metrics for risk stratification and personalized treatment planning in early AD.

## Methods

This retrospective, real-world observational report was based on data collected at the Cognitive Neurology Unit, Tel Aviv Medical Center. The cohort included 99 consecutive patients who initiated lecanemab treatment between November 2023 and June 2025. Seventeen patients were excluded because baseline MRI scans were not of sufficient quality for standardized quantitative analysis using the icobrain processing pipeline. In most cases, this was related to MRIs performed outside our institution with non-standardized acquisition protocols, missing required volumetric or susceptibility-weighted sequences, motion artifacts, incomplete datasets, or insufficient image resolution for reliable automated processing. These patients were therefore excluded from the imaging-analysis cohort, although they did receive clinical treatment with lecanemab. Eighty-two patients were included in the final cohort. Diagnostic confirmation required biomarker evidence of amyloid pathology, defined by either an abnormal CSF Aβ42/ p-tau181 ratio, or amyloid-positive PET imaging (Vizamyl™ (Flutemetamol F 18 Injection)). Cognitive stage was determined by experienced cognitive neurologists according to established diagnostic criteria for AD and MCI, integrating clinical interview, functional assessment, cognitive testing, and biomarker confirmation. Specifically, patients were classified according to the revised Alzheimer’s disease diagnostic framework and IWG criteria [11, 12]. Patients receiving active anticoagulation, those with > 4 cerebral microbleeds or ≥ 1 macrohemorrhage on baseline MRI, superficial siderosis, severe white matter disease or evidence of cerebral amyloid angiopathy-related inflammation (CAA-ri) and individuals with significant non-AD neurological conditions were excluded from treatment and therefore not included in this cohort [8]. In accordance with EMA and UK guidelines, APOE ε4 homozygotes were not treated with lecanemab [13]. All patients received lecanemab 10 mg/kg intravenously every two weeks, as per protocol [3]. MRI surveillance for ARIA was performed at 1, 2, 4, and 6 months after treatment initiation using standardized assessment criteria updated in the FDA label.

Patients with incomplete MRI processing were excluded. For cognitive outcome analyses, only patients who completed a 12-month MMSE assessment were included (*n* = 38).

Baseline demographic and clinical variables including age, sex, year of education, APOE genotype, vascular risk factors (hypertension, hyperlipidemia, diabetes mellitus type 2) and antiplatelet therapy were collected. Cognitive assessments included Mini-Mental State Examination (MMSE) at baseline and follow-up. The primary cognitive outcome was change in MMSE from baseline to 12 months (ΔMMSE).

The frequency of ARIA was recorded from clinical neuroradiology reports performed by trained specialists [10, 14]. Only clinically reported ARIA (ARIA-E and/or ARIA-H) were used for this report, and no automated or AI-based ARIA detection was performed. 

### MRI acquisition and volumetric processing

All MRI scans were acquired using a standardized institutional protocol [9] with AC-PC alignment on a 3T Siemens MAGNETOM Prisma scanner. The imaging protocol included high-resolution volumetric 3D T1-weighted sequences for automated volumetric analysis and susceptibility-weighted imaging (SWI) sequences for assessment of cerebral microhemorrhages (MH) and superficial siderosis. Baseline MRI examinations included gadolinium administration when clinically indicated, whereas follow-up ARIA surveillance scans were performed without contrast. To ensure longitudinal consistency, the same institutional MRI acquisition protocols and sequence parameters were used across baseline and follow-up visits whenever feasible. MRI analyses in this study were based on the baseline MRI. MRI volumetric measurements were processed centrally using icobrain dm (version 5.16.6, icometrix NV, Leuven, Belgium), a clinically validated and FDA-cleared automated analysis pipeline. Extracted measures included whole-brain, gray-matter (GM), white-matter (WM), regional cortical GM (frontal, temporal, parietal, occipital), hippocampal volume, and inferior lateral ventricular (ILV) volume, all normalized for head size. Age and sex-adjusted percentile scores for each structure were calculated by comparing each structure’s measurement to a large pool of reference data from a normative population. The percentile score of the ILV-to-hippocampus ratio served as an index of relative hippocampal atrophy This ratio has previously been validated as an automated surrogate marker of hippocampal neurodegeneration and is integrated within the icobrain workflow used in our study [15]. Baseline cerebral MH burden was assessed in susceptibility-weighted imaging (SWI) by expert neuroradiologist visual reading and by automated icobrain dm counts with manual refinement by the icometrix team. The version of the icobrain dm MH detection model used in this study was originally trained and validated primarily on GRE T2*-weighted MRI sequences. Because SWI sequences were preferentially used in this real-world cohort due to their higher sensitivity for detecting microhemorrhages and superficial siderosis, an additional manual quality-control refinement step was incorporated into the icometrix analysis pipeline. This process consisted of targeted review of algorithm-identified lesion candidates to exclude obvious false-positive detections and confirm uncertain findings, rather than full manual rereading of scans. The refinement process was performed independently and blinded to the neuroradiological assessments conducted by the Tel Aviv Medical Center clinical team. To assess concordance between the two measurement methods, we calculated the Intraclass Correlation Coefficient (ICC) using a two-way mixed-effects model with absolute-agreement criteria. 

### Predictors of cognitive response

Three hypothesis-driven linear regression models were used to assess baseline MRI predictors of ΔMMSE at 12 months. Each model evaluated one a priori MRI region of interest (ROI): GM volume, WM volume, or hippocampal volume (all three normalized for head size).

These ROIs were selected based on their established relevance to neurodegeneration and cognitive decline in AD [16–19] while limiting the number of tested imaging variables in order to reduce overfitting and multiple-comparison bias in this modest-sized real-world cohort. Age, sex, and baseline MMSE score were included as additional confounding predictors. Multiple comparisons were controlled using the Benjamini-Hochberg false discovery rate (FDR) method across the three ROI models.

To identify baseline predictors of ARIA occurrence (defined as ≥ 1 episode of ARIA-H, ARIA-E, or mixed ARIA-E/H), we performed two separate binary logistic regression analyses. ARIA rates were calculated across the entire cohort, as all patients had at least 6 months of follow-up and completed all protocol-specified MRIs. Baseline predictors included APOE ε4 carrier status (yes/no), hypertension (yes/no), antiplatelet use (yes/no), and either the human-rated MH count or the icometrix-rated MH count.

Connected MH identified by icometrix were counted as 1, to ensure consistency with the clinical protocol. The dependent variable was the presence of any ARIA at any follow-up MRI (yes/no), defined according to neuroradiology reports. Odds ratios (OR), 95% confidence intervals, and p-values were reported, and overall model fit was evaluated using the Wald χ² statistic. 

### Statistical analysis

Continuous variables were summarized using means and standard deviations; categorical variables as frequencies and percentages. Linear regression models report unstandardized coefficients (B), standardized regression coefficient (β), standard errors (SE), and p-values. Logistic regression models report ORs with 95% confidence intervals. Analyses were conducted using SPSS version 29.0.2.0 (20) (IBM Corp.). The study was approved by the Institutional Review Board of Tel Aviv Medical Center (0251-24-TLV).

## Results

Eighty-two patients were included in the baseline analysis. The mean baseline MMSE was 24.1 ± 2.8; 82.9% met criteria for MCI and the remainder for mild dementia. Mean age at baseline MRI was 72.6 ± 8.0 years, and 51.2% were female. 58.5% were APOE ε4 carriers. Hypertension was present at 47.5%, hyperlipidemia in 57.3%, diabetes mellitus in 17.1% and 35.4% were taking antiplatelet therapy. Sixty-six patients (80.5%) underwent CSF testing, 16 patients (19.5%) underwent amyloid PET imaging, and 2 patients (2.4%) had both modalities performed. (full baseline characteristics are detailed in Table 1).

**Table 1:** Characteristics of the study population

Characteristic	Value*
Baseline (*N* = 82)	
Age, years	72.6 ± 8.0 (45.4–85.2)
Female sex	42 (51.2%)
Years of education (*n* = 78)	15.1 ± 2.9 (8–23)
APOE ε4 carrier	48 (58.5%)
Hypertension	39 (47.5%)
Hyperlipidemia	47 (57.3%)
Type 2 diabetes	14 (17.1%)
Antiplatelet use	29 (35.4%)
Baseline cognitive status	
Mild cognitive impairment	68 (82.9%)
Mild dementia	14 (17.1%)
Moderate dementia	0
MMSE score at baseline (*n* = 81)	24.1 ± 2.8 (18–29)
CSF testing	66 (80.5%)
Amyloid PET	16 (19/5)
Follow-up (12 months) (*N* = 44)	
MMSE score at 12 months	21.8 ± 5.8 (6–30)
MMSE change from baseline	−2.5 ± 5.1 (− 17 to + 6)
Cognitive status at 12 months	
Mild cognitive impairment	25 (56.8%)
Mild dementia	16 (36.4%)
Moderate dementia	3 (6.8%)
ARIA outcomes (*N* = 82)	
Any ARIA (E or H)	17 (20.7%)
ARIA-H only	12 (14.6%)
Microhemorrhages	10 (12.2%)
Superficial siderosis	2 (2.4%)
ARIA-E only	1 (1.2%)
Mixed ARIA-E and ARIA-H	4 (4.9%)

*Values are presented as mean ± SD or n (%)

MRI-derived structural measures (icometrix) demonstrated a mean GM percentile of 8.6 ± 13.3 and mean hippocampal percentile of 15.9 ± 19.5. Ventricular enlargement relative to hippocampal size was markedly elevated, with an ILV/hippocampus ratio percentile of 86.3 ± 22.8 (full MRI-based measures are shown in Table 2), indicating significant hippocampal atrophy.

**Table 2:** MRI-based volumetric and microhemorrhage measures at baseline (*N* = 82)

MRI measure	Value*
Microhemorrhages	
Microhemorrhage count (human read)	0.26 ± 0.56 (0–3)
Microhemorrhage count (icometrix)	0.30 ± 0.56 (0–3)
Whole brain volumes	
Whole brain normalized volume	1374.7 ± 61.8 (1258.6–1536.3)
Whole brain volume percentile	11.5 ± 16.3 (0–68.9)
Gray matter volumes	
Gray matter normalized volume	789.9 ± 44.0 (659.3–867.9)
Gray matter volume percentile	8.6 ± 13.3 (0–51.5)
White matter volumes	
White matter normalized volume	584.8 ± 41.9 (503.8–686.9)
White matter volume percentile	38.8 ± 30.5 (0.2–100)
Regional gray matter volumes	
Temporal lobe GM normalized volume	125.3 ± 11.3 (99.8–150.0)
Temporal lobe GM percentile	11.2 ± 19.0 (0–86.1)
Frontal lobe GM normalized volume	203.9 ± 13.9 (170.8–234.1)
Frontal lobe GM percentile	11.9 ± 16.6 (0–74.5)
Parietal lobe GM normalized volume	132.6 ± 9.5 (104.3–153.2)
Parietal lobe GM percentile	6.9 ± 11.3 (0–52.5)
Occipital lobe GM normalized volume	66.0 ± 6.8 (51.6–79.6)
Occipital lobe GM percentile	27.5 ± 25.5 (0–85.6)
Hippocampal measures	
Hippocampal normalized volume	7.78 ± 0.99 (5.14–9.74)
Hippocampal volume percentile	15.9 ± 19.5 (0–88.2)
Ventricular measures	
ILV/hippocampus ratio	57.4 ± 30.9 (15.6–139.5)
ILV/hippocampus ratio percentile	86.3 ± 22.8 (18.4–100)

*Values are presented as mean ± SD or n (%)

**Fig. 1:**
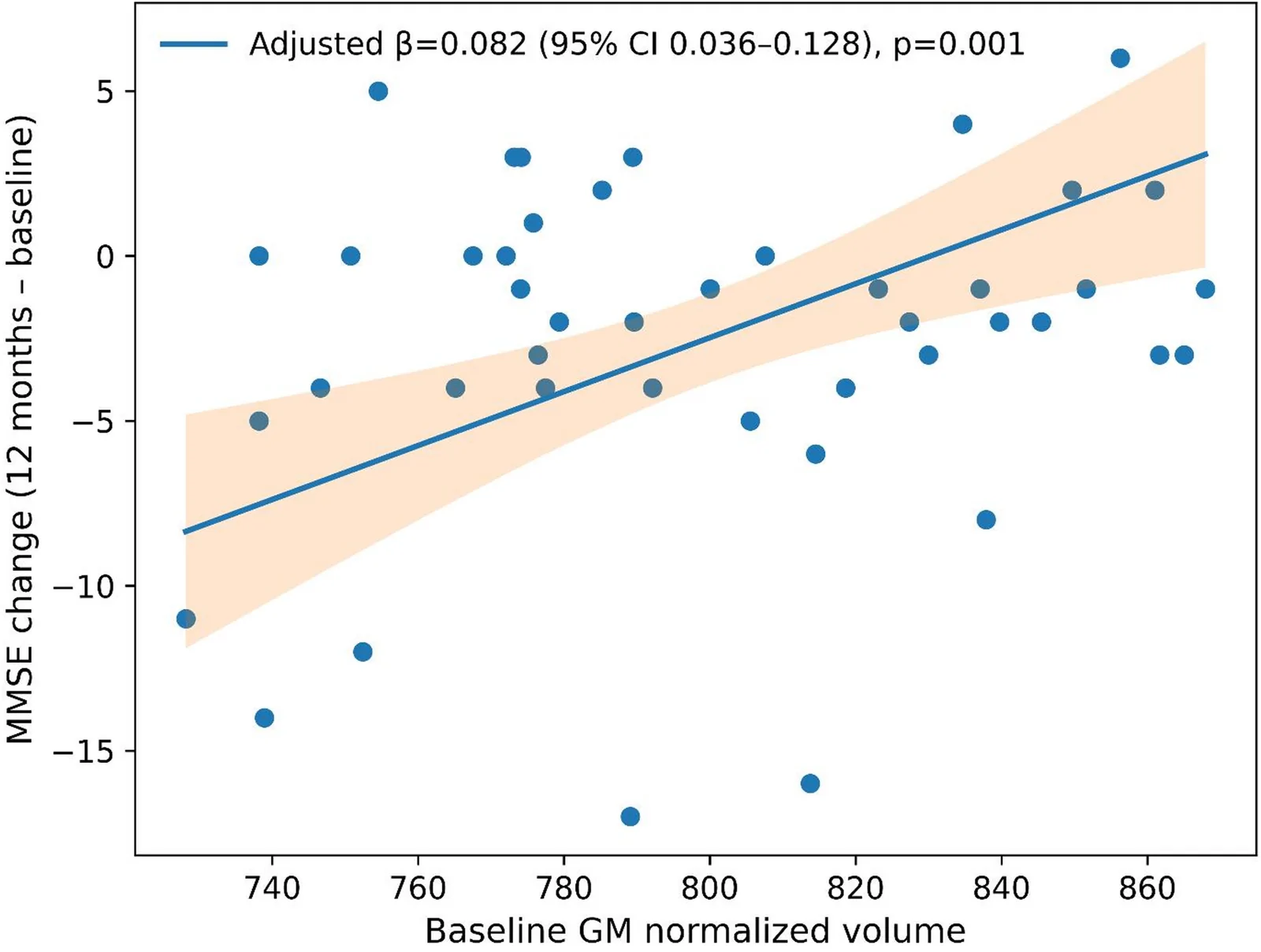
Association between baseline GM volume and change in MMSE at 12 months. Association between baseline global gray matter (GM) normalized volume and change in MMSE over 12 months. The regression line represents estimates from a linear model adjusted for age at baseline MRI, sex, and baseline MMSE. Higher baseline GM volume was independently associated with less MMSE decline (β = 0.082, 95% CI 0.036–0.128, p = 0.001)

Forty-four patients completed a 12-month cognitive assessment. The mean (± SD) and median change in MMSE from baseline to 12 months were − 2.5 (± 5.1) points and − 2.00, respectively. Among baseline MCI patients, 12/34 (35.3%) progressed to dementia over 12 months. Among baseline mild dementia patients, 1/4 (25.0%) progressed to moderate dementia. Higher baseline normalized GM volume was significantly associated with less MMSE decline in 12 months (B = 0.082, SE = 0.023, β = 0.639, t = 3.606, *p* < 0.001, FDR-corrected *p* < 0.003) (Fig. 1). Older age at baseline was also independently associated with less MMSE decline over 12 months (B = 0.391, SE = 0.105, β = 0.604, *p* < 0.001). In contrast, baseline normalized WM volume was not significantly associated with cognitive change (B = − 0.030, SE = 0.021, β = −0.232, *p* = 0.161), nor was normalized hippocampal volume (B = − 0.127, SE = 1.040, β = −0.022, *p* = 0.904).

Baseline microhemorrhage (MH) counts ranged from 0 to 3 for both human-rated and icometrix-derived measures, with mean values of 0.26 ± 0.56 and 0.30 ± 0.56, respectively. Agreement between human and AI-assisted MH ratings was excellent, with a single-measure ICC of 0.89 (95% CI: 0.83–0.93). A total of 17 out of 82 patients (20.7%) developed ARIA during treatment. One patient initially developed asymptomatic moderate ARIA-E detected on routine MRI surveillance, which progressed days later to severe ARIA-E accompanied by mild confusion. The patient was hospitalized and treated with intravenous corticosteroids for 5 days, with complete clinical recovery within approximately one week.

Lecanemab was permanently discontinued. Apart from in this patient, all other ARIA events were asymptomatic. In the first multivariable logistic regression model, which included human-rated MH count, hypertension, antiplatelet use, and APOE Ɛ4 carrier status, human-rated MH count emerged as a significant predictor of ARIA (B = 1.24, SE = 0.51, Wald = 5.89, *p* = 0.01), corresponding to an odds ratio (OR) of 3.48. Hypertension (OR = 1.07, *p* = 0.91) and antiplatelet use (OR = 2.02, *p* = 0.26) were not associated with ARIA risk, while APOE Ɛ4 carrier status showed a nonsignificant trend toward increased odds (OR = 3.37, *p* = 0.07). In a parallel model substituting icometrix-rated MH count, the association also reached statistical significance (B = 1.18, SE = 0.50, Wald = 5.51, *p* = 0.01), corresponding to OR of 3.25. Hypertension (OR = 1.15, *p* = 0.82), antiplatelet use (OR = 1.79, *p* = 0.35), and APOE ε4 carrier status (OR = 2.95, *p* = 0.10) did not reach statistical significance.

## Discussion

We found that in this real-world cohort of lecanemab-treated patients, higher normalized gray-matter (GM) volume at treatment initiation was the strongest predictor of a more favorable 12-month cognitive trajectory, even after adjustment for key covariates. As expected, GM volume declined with age.

Nevertheless, baseline GM volume remained independently associated with less cognitive decline, showing that lower cerebral volumes at treatment initiation still meaningfully predicts outcome.

AD is characterized by global cortical atrophy with regionally selective involvement of the medial temporal lobe, most prominently reflected by hippocampal volume loss [20]. However, our findings indicate that overall GM volume is more closely associated with subsequent cognitive trajectories thanWM and hippocampal volume alone. While longitudinal neuroimaging studies have consistently shown that progressive structural MRI changes correlate with cognitive and functional decline [21–25] our results emphasize the prognostic importance of baseline neurodegeneration in predicting treatment outcomes. These observations are concordant with accumulating evidence that lecanemab is most effective in individuals at earlier biological stages of disease and with relatively preserved brain structure [18, 26, 27]. Similarly, treatment with donanemab, another FDA-approved amyloid-targeting therapy, has demonstrated greater efficacy in patients with milder cortical atrophy and lower tau burden [28–30]. In this cohort hippocampal and white-matter volumes did not predict treatment response.

Several factors may explain this. Our cohort included patients with atypical AD presentations, in whom hippocampal atrophy may be less pronounced relative to diffuse cortical loss. In addition, many patients had severe hippocampal volume loss at baseline, creating a potential “floor effect” and limiting variability.

Notably, even though most patients in the cohort were defined clinically as MCI, GM volumes were found to be in the lowest population percentiles, indicating advanced neurodegeneration at treatment initiation, despite clinical stage. The relatively high educational attainment in this group may partially explain this mismatch and is consistent with the cognitive reserve theory [31], however this will need to be explored further in other real world cohorts.

The overall ARIA rate in our cohort was 20.7%, most were isolated MH (58.8% of total ARIA), and only one patient with ARIA-E was symptomatic aligning with results of CLARITY-AD and the open-label extension study [32]. Importantly, the use of the SWI sequence, which is substantially more sensitive than T2* weighted gradient echo in detecting cerebral microbleeds, likely increased identification of ARIA-H events that may be missed using less sensitive sequences [33]. Baseline MH burden emerged as the dominant predictor of ARIA, with each additional MH at baseline increasing the odds of ARIA by roughly 3.5-fold. This is clinically meaningful, given that patients with > 4 MH were excluded from treatment, highlighting that even within the eligible range, microvascular fragility remains a significant risk factor for ARIA. This finding aligns with previous review and guidelines published [6, 34]. APOE ε4 carriage showed the expected trend toward increased risk of ARIA [35], though did not reach significance, likely due to sample size constraints and the exclusion of APOE ε4 homozygotes. In this cohort, antiplatelet therapy and hypertension were not associated with ARIA. Previous studies have shown that elevated blood pressure, specifically higher mean arterial pressure, is associated with an increased risk of ARIA, particularly ARIA-E, in patients being considered for ATTs, and the use of antihypertensive medication was independently associated with a decreased risk of ARIA-E [14, 36, 37]. Antiplatelet therapy does not appear to significantly increase the overall risk of ARIA in patients receiving ATTs, but may contribute to more severe hemorrhagic events in high-risk individuals [35, 36, 38]. Ongoing safety data collection remains essential for optimal management.

A distinctive feature of our cohort is the integration of standardized AI-based volumetric MRI processing through collaboration with icometrix. Automated extraction of whole-brain, regional gray-matter, and hippocampal volumes, as well as age-adjusted percentile scoring, enabled high-resolution characterization of brain structure and contributed to the identification of imaging markers associated with clinical trajectories during treatment. Importantly, as this was an observational treated cohort without an untreated comparator group, these findings should not be interpreted as treatment-specific efficacy markers, but rather as markers associated with cognitive trajectory during treatment. MH count at baseline, which was found to be crucial for ARIA risk assessment, was also found to be accurate in an AI-assisted rating process. ICC analysis demonstrated excellent agreement between human and AI-assisted MH ratings, indicating that AI-supported MH quantification provides measurements that are highly consistent with expert human assessment and similarly informative with respect to ARIA risk.

These findings support the potential role of AI-based tools into clinical decision-making, with the potential to enhance scalability, efficiency, and standardization of microhemorrhage assessment across clinical settings.

This study has several limitations. The sample size for 12-month cognitive outcomes was modest, limiting power to detect weaker associations. Three ROIs were selected based on their established biological and clinical relevance in AD. We acknowledge that future studies with larger cohorts should aim to incorporate other brain regions, specifically, global ventricular measures and regional ventricular metrics, which may provide complementary information. Only MMSE was available as a cognitive endpoint, and more comprehensive neuropsychological measures may provide better sensitivity and allow for better assessment of cognitive reserve measures. In addition, ARIA was defined by clinical reads, without AI-based or volumetric ARIA detection. Regarding the AI-assisted MRI analysis pipeline, the icobrain dm MH detection model used in this study was primarily developed for GRE T2*-weighted MRI sequences, whereas SWI sequences were preferentially used in our cohort because of their higher sensitivity for ARIA-related findings. Consequently, manual refinement of AI-generated lesion candidates was required for SWI-based analysis. Although these refinements were performed independently and blinded to expert neuroradiological assessments, the results should be interpreted as reflecting an AI-assisted rather than fully autonomous workflow, consistent with current real-world clinical implementation.

In conclusion, our findings demonstrate the feasibility and utility of incorporating AI-based neuroimaging into routine ATT workflows, and feasibility of integrating AI-driven neuroimaging into ATT workflows. These findings also highlight the importance of imaging based biological staging to identify patients most likely to benefit from ATTs and those most at risk of side effects. 

## Data Availability

No datasets were generated or analysed during the current study.
